# Annual acknowledgement of reviewers

**DOI:** 10.1186/s12867-015-0032-x

**Published:** 2015-05-14

**Authors:** Tim Sands

**Affiliations:** BioMed Central, Floor 6, 236 Gray’s Inn Road, London, WC1X 8HB UK

## Abstract

The editors of BMC Molecular Biology would like to thank all our reviewers who have contributed to the journal in Volume 15 (2014).

**Samit Adhya**

India

**Simak Ali**

United Kingdom

**Randy Allen**

USA

**Paola Amable**

Brazil

**Emira Ayroldi**

Italy

**Veronique Baron**

USA

**Niall Barron**

Ireland

**Olivier Becherel**

Australia

**Agnieszka Bialkowska**

USA

**James Bieker**

USA

**Edward Bolt**

United Kingdom

**Matthew Brook**

United Kingdom

**Jorg Bungert**

USA

**Wu Cai**

China

**Giordano Calloni**

Brazil

**Adelino Canario**

Portugal

**Beatrice Cardinali**

Italy

**John Chan**

Canada

**Che-Chang Chang**

Taiwan

**Seong-Jun Cho**

South Korea

**Terrance Cooper**

USA

**Anita Corbett**

USA

**John Dahl**

Norway

**Paul Delgado Olguin**

Canada

**Isabelle Duluc**

France

**David Edgell**

Canada

**Bin Fan**

China

**Mark Feinberg**

USA

**Zhiyong Feng**

China

**Trine Fink**

Denmark

**Homa Ghalei**

USA

**Adriana Girardi**

Brazil

**Andreas Gruber**

Switzerland

**Jiannan Guo**

USA

**M. R. Hajimorad**

USA

**Alfred Handler**

USA

**Tokuko Haraguchi**

Japan

**Rocio Hassan**

Brazil

**Qihong Huang**

USA

**Yen-Sung Huang**

Taiwan

**Olivier Humbert**

USA

**Jan-Jong Hung**

Taiwan

**Gyorgy Hutvagner**

Australia

**Bulent Icgen**

Turkey

**Mukesh Jain**

USA

**Yuqian Jiang**

USA

**Shyan-Yuan Kao**

USA

**Fatah Kashanchi**

USA

**Hyungjin Kim**

USA

**Jeong-Sun Kim**

South Korea

**Robert Klopfleisch**

Germany

**Ajay Kohli**

Philippines

**Filip Lankas**

Czech Republic

**Seong-Ho Lee**

USA

**Pablo Liddle**

Uruguay

**Ling-Zhi Liu**

USA

**Birgit Lohberger**

Austria

**Sylvain Loric**

France

**Laura Lossi**

Italy

**Long Ma**

United Kingdom

**Li Ma**

USA

**Aida Martinez-Sanchez**

United Kingdom

**Gunter Meister**

Germany

**Lourdes Mengual**

Spain

**Marija Mihailovich**

Italy

**Christen Mirth**

Portugal

**Jan Mol**

Netherlands

**Hector Musto**

Uruguay

**Akira Muto**

Japan

**Hannu Myllykallio**

France

**Satoshi Nakajima**

USA

**Norbert Nass**

Germany

**Nicolas Negre**

France

**Zhongfu Ni**

China

**Peter Olson**

United Kingdom

**Kamil Önder**

Austria

**Tanya Parish**

USA

**Yoonseong Park**

USA

**Nagarekha Pasupuleti**

USA

**Andor Pivarcsi**

Sweden

**Barry Powell**

Australia

**Stéphane Ravanel**

France

**Félix Recillas-Targa**

Mexico

**Nicholas Redshaw**

United Kingdom

**Derek Richard**

Australia

**Judith Roe**

USA

**Remo Rohs**

USA

**Jacob Seeler**

France

**Joann Sekiguchi**

USA

**Gregory Shipley**

USA

**Hideaki Shiraishi**

Japan

**Rosalia Simmen**

USA

**Radek Sindelka**

USA

**Chris Smith**

United Kingdom

**Gillian Smith**

United Kingdom

**Gustavo Somoza**

Argentina

**Travis Stracker**

Spain

**Henk Stunnenberg**

Netherlands

**Per Sunnerhagen**

Sweden

**Minoru Takase**

Japan

**Gerald Thiel**

Germany

**Chenxi Tian**

USA

**Eric Toth**

USA

**Maite Vaslin**

Brazil

**Jesper Troelsen**

Denmark

**Pablo Vivas**

Puerto Rico

**Sonja Maria Walzer**

Austria

**Ju-Ming Wang**

Taiwan

**Chunyao Wei**

USA

**Ulrich Friedrich Wellner**

Germany

**Laurence Wurth**

Spain

**Zhaohui Xu**

USA

**Poliane Zerbini**

Brazil

**Chunyi Zhang**

China

**Man Zhou**

USA

**Suwen Zhu**

China

**Victor Zhurkin**

USA

